# Mild Potassium Chloride Stress Alters the Mineral Composition, Hormone Network, and Phenolic Profile in Artichoke Leaves

**DOI:** 10.3389/fpls.2016.00948

**Published:** 2016-06-28

**Authors:** Luigi Lucini, Daniela Borgognone, Youssef Rouphael, Mariateresa Cardarelli, Jamila Bernardi, Giuseppe Colla

**Affiliations:** ^1^Institute of Environmental and Agricultural Chemistry, Università Cattolica del Sacro Cuore, PiacenzaItaly; ^2^Department of Agricultural and Forestry Sciences, University of Tuscia, ViterboItaly; ^3^Department of Agricultural Sciences, University of Naples Federico II, NaplesItaly; ^4^Consiglio per la Ricerca in Agricoltura e l’Analisi dell’Economia Agraria, Centro di Ricerca per lo Studio delle Relazioni tra Pianta e Suolo, RomaItaly; ^5^Department of Sustainable Crop Production, Università Cattolica del Sacro Cuore, PiacenzaItaly

**Keywords:** *Cynara cardunculus* subsp. *scolymus*, flavonoid conjugates, lipid peroxidation, metabolomics, phytohormones, salinity

## Abstract

There is a growing interest among consumers and researchers in the globe artichoke [*Cynara cardunculus* L. subsp. *scolymus* (L.) Hegi] leaf extract due to its nutraceutical and therapeutic properties. The application of an abiotic stress such as salinity can activate the stress-signaling pathways, thus enhancing the content of valuable phytochemicals. The aim of this study was to assess the metabolic changes in artichokes by probing the leaf metabolome of artichoke plants grown in a floating system and exposed to a relatively mild (30 mM) potassium chloride (KCl) salt stress. Potassium chloride treatment decreased the leaf dry biomass of artichoke, macro- and microelements in leaves (e.g., Ca, Mg, Mn, Zn, and B) but increased the concentrations of K and Cl. Metabolomics highlighted that the hormonal network of artichokes was strongly imbalanced by KCl. The indole-3-acetic acid conjugates, the brassinosteroids hormone 6-deoxocastasterone, and even more the cytokinin precursor N^6^-(Delta-2-isopentenyl)-adenosine-5′-triphosphate, strongly increased in leaves of KCl-treated plants. Moreover, KCl saline treatment induced accumulation of GA_4_, a bioactive form additional to the already known GA_3_. Another specific response to salinity was changes in the phenolic compounds profile, with flavones and isoflavones being decreased by KCl treatment, whereas flavonoid glycosides increased. The osmotic/oxidative stress that salinity generates also induced some expected changes at the biochemical level (e.g., ascorbate degradation, membrane lipid peroxidation, and accumulation of mannitol phosphate). These latter results help explain the molecular/physiological mechanisms that the plant uses to cope with potassium chloride stress exposure.

## Introduction

In recent years, the use of natural compounds, in particular plant secondary metabolites, has gained tremendous interest in various industrial sectors and among consumers worldwide (Bart and Pilz, 2011). Plants produce at least 200,000 distinct secondary metabolites, and some of these are of pharmaceutical and/or nutraceutical importance (Pichersky and Gang, 2000; Bourgaud et al., 2001; Menin et al., 2010). Therefore, industrial sectors (i.e., food, cosmetic, and pharmaceutical) are progressively orientated toward plant-based compounds to replace the conventional synthetic chemicals, which can be harmful to the environment as well as to human health due to their suspected carcinogenicity (Velioglu et al., 1998).

The Asteraceae (ex Compositae) family has great socio-economic value, as it includes several species of horticultural interest, such as *Cynara cardunculus*. The globe artichoke [subsp. *scolymus* L. (Hegi)] is a dietary and medicinal plant species cultivated worldwide for its immature inflorescence known as “capitula” or heads, which have an edible receptacle and bracts (Lattanzio et al., 2009; de Falco et al., 2015). Artichoke leaves are also utilized as a raw material for the production of food additive-preservatives, nutraceuticals, cosmetics, and medical drugs (Lattanzio et al., 2009; de Falco et al., 2015). Several epidemiological and pharmacological studies have demonstrated the health promoting effects of artichoke extracts, including its antimicrobial, antioxidant, anti-carcinogenic, anti-HIV, hepatoprotective, and urinative activities (Gebhardt and Fausel, 1997; Brown and Rice-Evans, 1998; Noldin et al., 2003; Zhu et al., 2004; Falleh et al., 2008; Kammoun et al., 2010). These nutraceutical properties of artichoke leaves have been mostly attributed to their special chemical composition, in particular the high concentrations of polyphenols and inulins. Neochlorogenic acid (5-*O*-caffeoylquinic acid), 1,5- and 3,4-di-*O*-caffeoylquinic acids, and cynarin are the predominant compounds among the hydroxycinnamates, whereas, the major flavonoids are conjugated apigenin and luteolin (Lattanzio et al., 2009; Pandino et al., 2010, 2011, 2015).

Floating raft culture, widely used for leafy vegetable production, can represent an alternative to traditional soil-based culture of leaf artichoke, as it offers the possibility to maximize productivity, shorten the growing cycle with year-round production, and grow plants at high density (Tomasi et al., 2015). Moreover, the floating cultivation system provides the opportunity to enhance and/or modify the content of bioactive secondary metabolites through proper management of nutrient solution composition/concentration (Fallovo et al., 2009a,b; Tomasi et al., 2015).

The application of a mild stress such as salinity or nutrient stress can activate stress-signaling pathways, leading to an increase in the content of valuable phytochemicals (Colla et al., 2013a; Borgognone et al., 2014). Under abiotic stress conditions, plants activate a set of counteracting measures (e.g., physiological and molecular mechanisms) that allow adaptation to a sub-optimal environment (Atkinson and Urwin, 2012). Among these measures, the biosynthesis and accumulation of secondary metabolites (i.e., phenols, tocopherols, polyamines, and glucosinolates) with multiple functions (e.g., reactive oxygen species scavenging) play a crucial role in ensuring plant growth under adverse conditions (Lucini et al., 2015). More interestingly, these molecules are also important for human health, and therefore they indirectly attribute an added value (over that of non-stressed plants) to the basic nutraceutical properties of plants (Rouphael et al., 2012a). It has been shown that, by increasing salinity (e.g., 30 mM NaCl) in a floating system, it is possible to improve secondary metabolites in the artichoke spp. leaves (Colla et al., 2013a). Similarly, Borgognone et al. (2014) evaluate the effect of three chloride salts (CaCl_2_, KCl, and NaCl) at equimolar concentrations on biomass quality of artichoke and *C. cardunculus* (cardoon) growing in a floating raft system. The authors demonstrated that the application of KCl was more effective than NaCl and CaCl_2_ in enhancing total phenols, flavonoids, antioxidant activity, and target polyphenols such as luteolin and cynarin at the first leaf harvest.

Although, the complex network of metabolites is drastically altered under salt stress conditions (Ramalingam et al., 2015), previous studies (Colla et al., 2013a; Borgognone et al., 2014) focused only on few target compounds. In this regard, metabolomics provides an efficient tool for analyzing the developmental changes of metabolites at the whole-metabolome scale (Fukushima and Kusano, 2013; Farag, 2014). Over the last decade, metabolomic approaches have been increasingly used for elucidating regulatory networks involved in stress responses that allow the identification of potential biomarkers linked to improved stress tolerance (Fukushima and Kusano, 2013; Lucini et al., 2015; Witzel et al., 2015).

There is a growing interest among consumers and researchers on how the application of mild stresses can enhance the content of bioactive metabolites, although agronomic applications have often been hampered by a lack of published information on the physiological/molecular mechanisms determining artichoke response to KCl salt exposure. In this work, we report the effects of KCl application on yield and changes in the leaf metabolome of artichokes grown in a floating system, taking into account both the triggering effect toward health-promoting compounds and the biochemical changes involved in stress response.

### Materials and Methods

### Growth Conditions, Plant Material, and Nutrient Solution Composition

The experiment was carried out in a polyethylene greenhouse situated at the experimental farm of Tuscia University, central Italy (latitude 42°25′ N, longitude 12°08′ E, altitude 310 m). The daily air temperature of the greenhouse was maintained between 12 and 30°C and the day/night relative humidity was 55/80%.

Seeds of *C. cardunculus* subsp. *scolymus* (L.) Hegi ‘Romolo’ (La Semiorto Sementi, Lavorate di Sarno, Italy) were sown in polystyrene plug trays on March 21, 2012. At two-true-leaf stage (22 days after sowing), artichoke plants were moved to a floating raft cultivation system. The floating raft system consisted of the polystyrene plug trays floating in plastic tanks with a constant volume (60 L) of aerated nutrient solution. An air compressor maintained the dissolved oxygen content above 6 mg L^-1^. The plant density was 463 plants m^-2^, as used commercially in floating systems.

A randomized complete block-design with four replicates was used to compare two nutrient solutions: a non-salt control and a saline solution with KCl. Each experimental unit consisted of 84 plants (0.1815 m^2^) in a container filled with 60 L of aerated nutrient solution. The composition of the basic nutrient solution was 13 mmol L^-1^ NO_3_-N, 1 mmol L^-1^ NH_4_-N, 1.75 mmol L^-1^ S, 1.5 mmol L^-1^ P, 5 mmol L^-1^ K, 4.5 mmol L^-1^ Ca, 2 mmol L^-1^ Mg, 1 mmol L^-1^ Na, 1 mmol L^-1^ Cl, 20 μmol L^-1^ Fe, 9 μmol L^-1^ Mn, 0.3 μmol L^-1^ Cu, 1.6 μmol L^-1^ Zn, 20 μmol L^-1^ B, and 0.3 μmol L^-1^ Mo, with an electrical conductivity of 2.0 dS m^-1^. The saline nutrient solution had the same basic composition plus an additional 30 mM of KCl, giving an electrical conductivity value of 5.1 dS m^-1^. The pH of the nutrient solution in both treatments was 6.0 ± 0.3. The nutrient solutions were renewed weekly and prepared using deionized water.

### Leaf Biomass and Chlorophyll Content Determination

The leaves of the artichokes were mowed three times (45, 84, and 109 days after sowing) during the growing cycle. The harvest time was decided by monitoring the plant height in all plots. In particular, when the plant height reached 25 cm in one plot, leaves were harvested from all plots. The leaf tissues were dried in a forced-air oven at 60°C for 72 h for dry biomass and dry matter percentage. The leaf dry biomass was expressed in g plant^-1^. The dried leaf tissue was used for mineral analysis.

The Soil Plant Analysis Development (SPAD) index was recorded at the termination of the experiment (103 days after sowing). A portable chlorophyll meter (SPAD-502, Minolta Corporation, Ltd., Osaka, Japan) was used to measure the relative leaf chlorophyll concentration as a rational unit. Measurements were made at the central point of the leaflet between the midrib and the leaf margin. Twenty random readings per plot were taken and averaged to a single SPAD value for each treatment.

### Mineral Analysis

The dried leaf tissues of the artichokes were ground in a Wiley mill to pass through a 20-mesh screen, then 1 g samples were analyzed for the following macro and micronutrients: N, P, K, Ca, Mg, Cl, Fe, Mn, Zn, Cu, and B. Nitrogen (total N) concentration in leaf tissues was determined after mineralization with sulfuric acid (H_2_SO_4_, 96%, Carlo Erba Reagents, Cornaredo, Milan, Italy) in the presence of potassium sulfate (K_2_SO_4_) and a low concentration of copper (Cu) by the Kjeldahl method (Bremner, 1965). P, Ca, Mg, Cl, Fe, Mn, Zn, Cu, and B were determined by dry ashing at 400°C for 24 h, dissolving the ash in nitric acid (HNO_3_, 1:20 w/v) and assaying the solution obtained using an inductively coupled plasma emission spectrophotometer (ICP Iris, Thermo Optek, Milan, Italy; Karla, 1998). Chloride concentration was determined by titration with AgNO_3_ in the presence of K_2_CrO_4_ (Eaton et al., 1995).

### Metabolomic Analysis

Plant metabolites were determined through an omic approach using a 1290 ultra-high performance liquid chromatograph coupled to a G6550 quadrupole-time-of-flight mass spectrometer (MS; Agilent Technologies Santa Clara, CA, USA) via a Dual Electrospray JetStream ionization system (UHPLC/Q-TOF).

The analytical method was based on the work of Lucini et al. (2015), using an Agilent Zorbax Eclipse-plus column (75 mm × 2.1 mm i.d., 1.8 μm). Briefly, metabolites were extracted in 80% methanol (with 10 mM HCOOH) using an Ultra-Turrax, centrifuged, and then filtered using 0.2 μm syringe filters. Extracts were analyzed using reverse phase chromatography and gradient elution, with water and methanol serving as mobile phases. The injection volume was 2.5 μL and the MS was operated in MS mode, acquiring positive spectra in the range of 100–1200 m/z. Lock masses (m/z 121.0509 and 922.0098) were continuously infused in the ionization source through a dedicated electrospray, to achieve better mass accuracies.

Raw data were processed through a recursive analysis work flow, using Profinder B.05 (Agilent Technologies) for compounds features deconvolution and following alignment and filtering. Only the compounds that could be found in 100% of the replicates within at least one treatment were retained. The entire isotopic pattern (accurate mass, isotopic spacing, and isotopic ratio) of filtered features was then used for identification, against two custom databases: (i) the database exported from Phenol-Explorer 3.0 (Rothwell et al., 2013), and (ii) the database exported from PlantCyc 9.5 (Plant Metabolic Network^1^). A mass accuracy tolerance threshold of 5 ppm and an identification score above 80/100 were adopted as thresholds for identification. The identified compounds and their abundance (peak area) were finally exported for statistics.

### Statistical Analysis

The experimental data were subjected to one-way analysis of variance (ANOVA) using the software package SPSS 10 for Windows, 2001 (SPSS, Inc., Chicago, IL, USA). The metabolomic data were statistically elaborated using Mass Profiler Professional B.12.05 (Agilent Technologies). Abundances were log2 normalized and baselined against the median of each compound in the control samples. The ANOVA (*P* = 0.001, Bonferroni multiple testing correction) and fold-change analysis (cut-off = 5) were combined into volcano plots. Multivariate statistics were also applied to investigate actual compound profiles between control and treated samples: unsupervised cluster analysis (hierarchical cluster algorithm with similarity measure set to Euclidean) and principal component analysis (PCA) were carried out.

## Results

### Biomass Production, Leaf Dry Matter, and Chlorophyll Content

**FIGURE 1:**
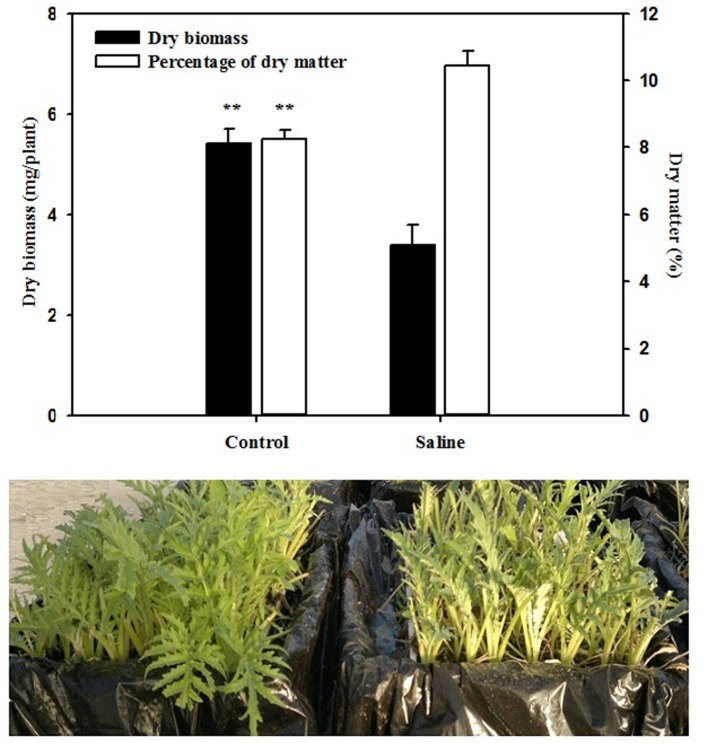
**Effect of salinity on total dry biomass and percentage of dry matter of artichoke leaves.** Bars represent standard errors. ^∗∗^*P* ≤ 0.01.

The total leaf biomass and leaf dry matter were significantly (*P* < 0.01) affected by salinity. The application of 30 mM KCl to the basic nutrient solution decreased the total biomass of artichoke plants by 37% in comparison with those recorded in the control treatment (**Figure 1**). An opposite trend was observed for the leaf dry matter percentage, which was positively affected by the application of KCl, with an increase of 26% over that of the non-salt treatment (**Figure 1**). Moreover, application of KCl resulted in a significant (*P* < 0.01; data not shown) increase in chlorophyll content, since the SPAD index was significantly higher by 19.2% in KCl (average 37.8) than the control artichoke plants (average 31.7).

### Mineral Composition

The macro and microelement concentrations in artichoke leaf tissue as a function of salinity are displayed in **Tables 1** and **2**. No significant difference among treatments was observed for the concentrations of N and P in leaves [average 35.3 and 8.6 g kg^-1^ dry weight (DW), respectively]. The application of KCl to the nutrient solution decreased the concentrations of Ca and Mg by 57.9 and 46.6%, respectively, from their concentrations in the non-salt treatment, whereas an opposite trend was observed for the K leaf concentration (**Table 1**). Moreover, the chloride concentration in artichoke leaf tissue was four-times higher in the KCl treatment compared to the non-salt one (**Table 1**).

**Table 1:** Effect of salinity on macronutrients and chloride (g kg^-1^ dry weight) in artichoke leaves.

Treatment	N	P	K	Ca	Mg	Cl
Control	35.4	8.6	44.2	12.6	3.0	16.7
Saline	35.2	8.6	49.5	5.3	1.6	62.3
Significance^a^	NS	NS	^∗^	^∗∗∗^	^∗∗∗^	^∗∗∗^

*^a^NS, ^∗^, ^∗∗∗^, non-significant or significant at *P* ≤ 0.05, and 0.001, respectively.*

**Table 2:** Effect of salinity on micronutrients (mg kg^-1^ dry weight) in artichoke leaves.

Treatment	Fe	Mn	Zn	Cu	B
Control	23.8	31.2	12.4	2.1	25.4
Saline	26.6	18.1	10.6	2.4	16.3
Significance^a^	NS	^∗∗∗^	^∗^	NS	^∗∗∗^

*^a^NS, ^∗^, ^∗∗∗^, non-significant or significant at *P* ≤ 0.05, and 0.001, respectively.*

The effect of KCl induced-salinity on tissue micronutrient concentrations (Mn, Zn, and B) was highly significant (**Table 2**). The application of KCl salt significantly reduced the concentrations of Mn, Zn, and B by 41.9, 51.6, and 35.8%, respectively, in comparison to the non-salt treatment, whereas no significant difference was observed for the concentrations of Fe and Cu in artichoke leaves (25.2 and 2.2 mg kg^-1^, respectively; **Table 2**).

### Metabolomic Profiling of Leaves

The combination of ultra-high performance liquid chroma tography (enabling higher chromatographic resolutions), quadrupole-time-of-flight high-resolution mass spectrometry, and comprehensive databases allowed a wide profiling of the phytochemicals in our extracts. All compounds were detected with good mass accuracies (nominally below 5 ppm, but in the sub-ppm range in several cases), while the alignment and filtering processes ensured reduction of false positives or compounds having weak confidences in identification.

**FIGURE 2:**
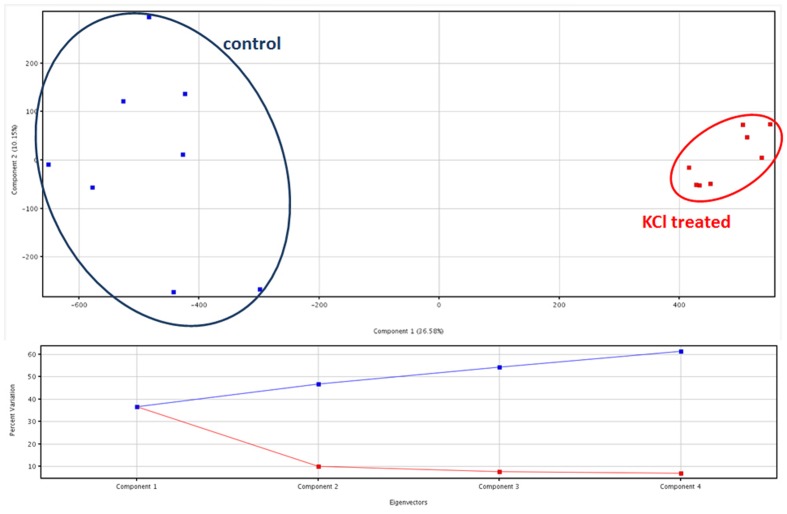
**Principal component analysis (PCA) of artichoke metabolites in KCl stressed and control plants, together with Eigenvectors for the percentage of variability explained**.

Principal component analysis provided two clearly separated clusters of samples, consistent with the corresponding treatment. Comparable results were provided from both the dataset on phenolic compounds (data provided as Supplementary Material) and the metabolites identified from PlantCyc (**Figure 2**). Coherently, unsupervised cluster analysis carried out from fold-change patterns (heat maps) resulted in the definition of two distinct clusters, one per treatment, each of them consisting of all replications within the treatment (Supplementary Material). Therefore, multivariate statistics provided clear evidence that differences between treatments were represented in our two datasets, thus supporting further investigations.

Differential compounds were selected from the processed datasets through Volcano plot analysis, where ANOVA and fold-change were combined (using a fold-change cut off = 3 and a *P*-value of 0.001). The compounds selected from the volcano plot analysis are summarized in **Tables 3** and **4** for the dataset on phenolic compounds and other plant metabolites, respectively.

**Table 3:** Phenolic metabolites resulting differential after Volcano analysis (*P*-value < 0.01, fold-change threshold = 3), together with individual *P*-values (Bonferroni multiple testing correction), regulation and fold change values.

Compound	*p* (Corr)	Regu lation	Fold-change
Apigenin	3.25E-04	Down	13.74
Chrysin	4.24E-05	Down	12.18

Luteolin-*O*-glucoside	1.93E-03	Up	3.86
Apigenin 6-*C-*glucoside 8-*C*-arabinoside	8.05E-03	Up	243.30

Genistein	3.25E-04	Down	13.74
Daidzein	4.24E-05	Down	12.18

Gallocatechin	7.71E-05	Up	8.76
Leucocyanidin	7.71E-05	Up	8.76
Quercitrin	1.93E-03	Up	3.86

Ferulic acid	4.45E-03	Down	3.05
1/4/5-Caffeoylquinic acid	1.99E-04	Down	10.27
3/4/5-Feruloylquinic acid	4.47E-03	Up	3.77
3-*p*-Coumaroyl-1.5-quinolactone	5.44E-04	Down	9.40

**Table 4:** Plant metabolites resulting differential after Volcano analysis (*P*-value < 0.01, fold-change threshold = 3), together with individual *P*-values (Bonferroni multiple testing correction), regulation and fold change values. Metabolites are grouped according to the chemical/physiological class they belong to.

Class	Compound	p (Corr)	Regulation	Fold-change
Carbohydrates	Mannitol-1-phosphate	1.68E-06	Up	10.82
	*D*-Gluconate	2.40E-03	Up	3.62
	*L*-Rhamnofuranose	1.82E-03	Up	3.01
	1,5-Anhydro-*D*-mannitol	1.82E-03	Up	3.01
	Beta-fucose 1-phosphate	1.01E-03	Up	992.96
	*S*-methyl-5-thio-*D*-ribose	3.79E-04	Down	1757.22

Hormones	Benzyladenine-7-*N*-glucoside	1.60E-04	Up	4.72
	Kinetin	5.03E-05	Up	3.68
	*N^6^-*(Delta^2^-isopentenyl)-adenosine 5’-triphosphate	2.43E-18	Up	16749.37
	1-Aminocyclopropane-1-carboxylate	1.38E-03	Up	2.30
	GA34-catabolite	1.27E-05	Up	3483.93
	GA29-catabolite	9.77E-04	Up	1140.86
	GA19	2.25E-03	Up	2.72
	GA36	2.26E-03	Up	2.72
	Gibberellin A20	9.53E-03	Up	225.29
	Gibberellin A8	4.04E-03	Down	821.79
	Gibberellin A4	9.52E-03	Up	234.27
	Gibberellin A9 methyl ester	4.48E-06	Down	7003.35
	Gibberellin A12	3.77E-22	Down	23014.80
	2-*cis*,4-*trans*-xanthoxin	4.68E-03	Up	2.27
	8′-hydroxyabscisate	5.73E-03	Up	562.88
	Indole-3-acetyl-leucine	1.63E-05	Up	2659.83
	Indole-3-acetyl-alanine	5.10E-07	Up	25.18
	12-oxo-*cis*-10.15-phytodienoate	8.75E-03	Up	2.37
	3-oxo-2-(*cis*-2’-pentenyl)-cyclopentane-1-octanoate	1.29E-06	Up	10.66
	12.13(*S*)-epoxylinolenate	8.72E-03	Up	2.36
	6-Deoxocastasterone	7.91E-06	Up	3601.39

Glucosinolates	8-Methylthiooctylhydroximoyl-cysteinylglycine	2.86E-05	Up	5.13
	8-Methylsulfinyloctyl glucosinolate	9.31E-03	Up	330.70
	5-Methylthiopentyldesulfoglucosinolate	2.83E-05	Up	4.93
	6-Methylthiohexylhydroximate	5.10E-07	Up	7.10
	2-Propenyl-glucosinolate	4.04E-03	Down	533.04

Lipids	18-Oxo-oleate	9.88E-03	Up	4.52
	9.10-EOT	8.72E-03	Up	2.36
	3-Oxohexanoyl-CoA	5.13E-20	Up	9486.33
	Eicosapentaenoyl-CoA	9.61E-04	Up	946.65
	9.10-EOD	1.30E-06	Up	10.67
	(9*Z*)-(13*S*)-12,13-epoxyoctadeca-9,11-dienoate	1.30E-06	Up	10.67
	Palmitoleate	6.21E-06	Up	6.41
	*N*-methylethanolamine phosphate	1.63E-04	Up	2.72
	*trans*-Delta^2^-decenoyl-CoA	9.77E-04	Up	1396.61
	1-Palmitoyl-2-linoleoyl-Phosphatidylcholine	7.35E-04	Down	5314.10
	CDP-choline	5.81E-16	Down	98001.02
	Phosphoryl-ethanolamine	8.33E-03	Down	636.28

Oxidative stress	Cyclic-2,3-*O*-oxalyl-*L*-threonate	1.07E-03	Up	1341.41
	*L*-idonate	2.40E-03	Up	3.62
	2-Carboxy-*L*-*threo*-pentonate	3.29E-16	Down	36165.31
	Dehydroascorbate (bicyclic form)	2.82E-03	Up	3.25
	4-Hydroxy-2-nonenal	1.10E-16	Up	15153.03
	*L*-Quinate	2.11E-05	Up	8.16

Terpenes	Geranyl-geranyl diphosphate	9.77E-04	Up	958.03
	Prephytoene diphosphate	3.42E-06	Up	6653.24
	2-*C*-methyl-*D*-erythritol-4-phosphate	5.10E-07	Up	7.66
	Retinal	3.77E-22	Up	9472.50
	Nonaprenyl diphosphate	4.11E-03	Down	793.31
	6-Methoxy-3-methyl-2-nonaprenyl-1,4-benzoquinol	1.94E-21	Up	8700.22
	Octaprenyl diphosphate	4.89E-06	Up	5861.76
	3-Demethylubiquinol-10	1.94E-21	Up	11970.13

Aminoacids	Methylacrylyl-CoA	1.62E-04	Up	4735.38
	(1*S*,2*R*)-1-*C*-(indol-3-yl)glycerol 3-phosphate	2.33E-05	Up	3.19
	Crotonyl-CoA	1.62E-04	Up	4735.38
	2-Oxo-3-phenylpropanoate	7.19E-04	Up	2.68
	*N*-acetyl-*L*-glutamate 5-semialdehyde	4.64E-03	Up	2.12
	2-Methylcrotonoyl-CoA	1.55E-05	Up	3762.58
	*L*-Phenylalanine	2.40E-04	Up	3.72
	*S*-methyl-*L*-methionine	1.09E-17	Down	56726.03
	Histidinol	2.70E-06	Up	4.71

Chlorophyll	7^1^-Hydroxychlorophyllide *a*	1.71E-04	Up	8275.37
	Uroporphyrinogen-III	1.56E-05	Up	3604.73
	Coproporphyrinogen III	8.32E-05	Up	2.73
	Dihydrogeranylgeranyl-chlorophyll *a*	6.95E-07	Down	2.22
	Coproporphyrinogen I	8.36E-05	Up	2.73
	Primary fluorescent chlorophyll Catabolite	5.46E-04	Up	2.96
	Red chlorophyll catabolite	5.42E-04	Up	2.96

Others	9-Alpha-copalyl diphosphate	9.77E-04	Up	912.53
	(-)-Medicarpin	8.76E-15	Down	38481.83
	*S*-Lactoyl-glutathione	3.12E-05	Up	5.86
	*L*-*erythro*-5,6,7,8-tetrahydrobiopterin	1.33E-03	Up	3.47
	6-Hydroxymethyl-dihydropterin Diphosphate	1.31E-05	Up	3691.44
	5-Hydroxyindole acetaldehyde	1.89E-18	Up	11476.60
	1-D-*myo*-inositol 1,2,3,4,5,6-hexakisphosphate	1.75E-05	Up	3572.08
	3,5-Bisdiphosphoinositol-D-*myo*-inositol (2,3,4,6)tetrakisphosphate	1.98E-21	Up	10687.16
	Acetoacetyl-CoA	2.27E-17	Up	43670.27
	Protocatechuate	4.89E-04	Up	2.46
	Purine	4.55E-03	Up	478.18
	*S*-adenosyl-4-methylthio-2-oxobutanoate	3.77E-22	Up	367851.94
	*S*-adenosyl-*L*-homocysteine	1.47E-03	Down	2.26
	*S*-(-)-ureidoglycolate	4.75E-12	Up	34887.25
	Dopamine	5.10E-07	Up	3.37
	Nicotinate	1.17E-06	Down	5.67
	Oxalyl-CoA	3.98E-04	Up	1753.74
	Dimethylsulfoniopropionate	3.83E-04	Down	1840.10
	Se-methyl-Se-methionine	4.89E-06	Down	12944.65
	3-Sulfopyruvate	4.00E-04	Down	2134.65

Regarding phenolics, a decrease was observed in the flavones apigenin and chrysin, and the isoflavones genistein and daidzein. Concurrently, the glucoside derivatives of the flavones luteolin and apigenin increased (namely luteolin-*O*-glucoside and apigenin-6-*C*-glucoside-8-*C*-arabinoside). Among other flavonoids, the flavans gallocatechin and leucocyanidin, together with the flavonol quercitrin, increased under the KCl treatment. Furthermore, most of the hydroxycinnamics decreased (ferulic acid, caffeoylquinic acid, and *p*-coumaroyl-1,5-quinonolactone) whereas feruloylquinic acid increased under the KCl treatment.

As expected, the number of metabolites resulting from the database generated via PlantCyc identifications was much higher than phenolics. Most of the metabolites could be related to secondary metabolism, and were classified for interpretations according to the biochemical class they belong to (**Table 4**).

The phytohormones profile was strongly imbalanced by the KCl treatment. A cytokinin precursor, N6-(Δ2-isopentenyl)-adenosine 5′-triphosphate, was highly accumulated under salinity conditions, as was the active cytokinin kinetin. This suggests that cytokinins are affected by the treatment, as indicated by the increase of benzyladenine 7-*N*-glucoside, an inactivation product (Hou et al., 2004). Coherently, the antagonistic hormone auxin [indole-3-acetate (IAA)] had an opposite trend. Two known storage forms, IAA-Ala and IAA-Leu, were both present during salinity conditions in leaves. Among all the plant hormones, GAs were the most affected by the treatment. Several GAs biosynthetic intermediates were involved, and most of them were greatly accumulated after treatment with KCl. GA12, the precursor of all GA forms, comprising the two main bioactive GAs, GA1 and GA4, had increased consumption under salinity conditions. In our experiments, both the pathways originating from GA12 were involved, i.e., the 13-hydroxylated pathway (GA19, GA20, GA29-catabolite, and GA8) and the main GA biosynthesis way (GA36, GA4, and GA34-catabolite). The bioactive form, GA4, was strongly accumulated after KCl treatment in leaves, together with other intermediates. Abscisic acid (ABA) metabolism was also involved, with the increased accumulation of the first intermediate of its degradation via the formation of phaseic acid (8′-hydroxyabscisate). Concurrently, the ABA precursor xanthoxin was slightly increased. Ethylene and brassinosteroid biosynthesis were only weakly altered by saline stress, with two intermediates increased. Jasmonate, a known stress-related hormone, did not change after treatment, although three early intermediates (12-oxo-10,15-phytodienate; 3-oxo-2-(*cis*-2′-pentenyl)-cyclopentane-1-octanoate; and 12,13-epoxylinoleate) increased under the salinity treatment. Finally, the ethylene precursor, 1-aminocyclopropane-1-carboxylate, was also increased by KCl. Besides plant hormones, a number of other leaf metabolites were altered in response to KCl treat ment. Among carbohydrates, mannitol derivatives, gluconate, rhamnose, and fucose phosphate accumulation was stimulated by KCl treatment, while only methyl-thio-ribose decreased. Glucosinolates biosynthesis was also triggered by KCl treatment, as well as terpene metabolism (including precursor units of quinols, geranylgeranyl diphospate, and octa- and nonaprenyl derivatives).

The amino acidic profile was altered by the treatment, even though a clear trend could not be identified: lysine, isoleucine, valine, and phenylalanine degradation products could be identified among differential metabolites, together with tryptophan, arginine, methionine, and histidine precursors. Other compounds, although not belonging to the same class, could be grouped because they were all related to oxidative stress. This class included ascorbate degradation products (2,3-oxalyl-*L*-threonate, *L*-idonate, 2-carboxy-*L*-threo-pentonate, and bicyclic dehydroascorbate) and the product of lipoperoxidation 4-hydroxy-2-nonenal. Concurrently, several metabolites related to membrane lipids could be identified. Among these, some oxidized forms (9,10-EOT, 3-oxo-hexanoyl-CoA and 9,10-EOD), together with free fatty acids and polar heads of phospholipids were increased under KCl stress. Choline phosphoglycerides and phosphoryl-ethanolamine were, however, reduced.

Potassium chloride treatment also induced the accumulation of chlorophyllide *a* (both 71-hydroxychlorophyllide a, and porphyrinogen derivatives increased), although the chlorophyll catabolites were higher in treated plants. Finally, there was some disparity between several other metabolites; however, they could not be grouped into specific biochemical classes. Among others, contrasting results were achieved for the phytoalexins medicarpin and α-copalyl diphosphate. Additionally, two bio pterin derivatives were stimulated, while *S*-adenosyl-*L*-homo cysteine, nicotinate, and some sulfur-containing compounds (i.e., dimethylsulfoniopropionate, Se-methyl-Se-methionine, and 3-sulphopyruvate) were decreased under KCl treatment.

## Discussion

The majority of vegetable species respond to elevated soil or water salinity by a decrease in plant growth and crop productivity due to Na^+^ and/or Cl^-^ induced inhibition of physiological and metabolic processes (Colla et al., 2010; Lucini et al., 2015). In this study, KCl application had a negative influence on the dry biomass production of artichokes (**Figure 1**) as demonstrated in other greenhouse studies on watermelon (Colla et al., 2006), zucchini squash (Rouphael et al., 2006), melon (Rouphael et al., 2012b), and cucumber (Colla et al., 2012, 2013b) grown hydroponically. According to Munns (2005), “plants display a two-phase growth response to salt stress conditions.” The first phase appears rapidly and is due to osmotic or water-deficit stress caused by salt outside the plants. The second phase takes time to develop, and results from the ion-excess or salt-specific effect of salt inside the plant, as the capacity of the cells to compartmentalize salt in the vacuole is exceeded (Epstein et al., 1980; Munns, 2005).

The macro- and microelement concentrations, recorded in the present study, indicate that artichokes entered the second phase stage because the application of KCl induced nutritional disorders (**Tables 1** and **2**). These disorders were associated with the excessive Cl^-^ uptake and accumulation in the leaves as well as the effect of salinity on nutrient availability, translocation, and accumulation within the plant (Grattan and Grieve, 1999; Dorais et al., 2001).

It has been generally accepted that the critical toxicity concentration of chloride in leaf tissues on a DW basis ranges from 4 to 7 g kg^-1^ and from 15 to 50 g kg^-1^ for Cl-sensitive and Cl-tolerant species, respectively (Marschner, 2012). In the current experiment, the chloride concentration in artichoke leaves recorded in the KCl treatment (e.g., 62.3 g kg^-1^ DW; **Table 1**) was beyond the critical threshold of 50 g kg^-1^ DW reported for salt-tolerant crops. Moreover, the high concentration of Cl^-^ in the external solution interferes with the uptake of other ions (e.g., Ca, Mg, Mn, Zn, and B; **Tables 1** and **2**). As a result, the artichokes became susceptible to nutritional disorders and chloride injury leading to reduced growth (Grattan and Grieve, 1999).

Moving into the metabolic changes induced by our specific KCl treatment (**Tables 3** and **4**), some results were expected, while some other interesting responses were found. Phytohormones have a prominent role in mediating plant response to abiotic stresses, and modulating physiological responses to help plants survive in adverse conditions. Many studies reported the involvement of plant hormones in response to soil salinity, and they are sometimes used to counteract the effect of salinity (Iqbal and Ashraf, 2013; Sharma et al., 2013). In this study, the biosynthesis of jasmonate, ethylene, ABA, IAA, GA, and cytokinin were involved in salt stress response, but some hormones had a prominent role. As a general consideration, our results suggest a response that differs from that induced by high NaCl concentrations as a result of different ion toxicity between K^+^ and Na^+^. Indeed, ABA, which is known to increase under abiotic stress conditions (as reviewed by Fahad et al., 2014), did not change in our experiments. The mitigation of salt stress from ABA involves a reduction in Na^+^ transport and Na^+^/K^+^ ratio (Gurmani et al., 2013). Possibly, ABA was not directly involved in the response to KCl because of the involvement of K^+^ and not Na^+^ in positive ions unbalance. Nonetheless, an inactivated form of ABA accumulated under higher salinity. Similar considerations can be made regarding jasmonic acid, which is only marginally involved in stress response, as deduced from the increase in the content of a few biosynthetic intermediates. Auxin homeostasis plays an important role in mediating plant growth in response to stress. Salt stress is known to decrease free IAA level under salinity conditions and to affect the expression of genes coding for enzymes responsible for IAA amide conjugation (Naser and Shani, 2016). The over-accumulation of two IAA amino acid conjugates after stress suggests that auxin balance might be involved in artichoke response to salinity, in agreement with the results reported in other crops (Naser and Shani, 2016). Despite the molecular and physiological role of IAA conjugates is not fully elucidated yet, their involvement in salt stress response has been confirmed in artichoke.

Cytokinin content was not reduced in our saline treatment, possibly because this hormone is involved in early response to salt stress (Nishiyama et al., 2012). Moreover, the involvement of GAs in contrasting salinity stress was observed. It is reported that bioactive GAs decrease after several biotic stresses; indeed, salt stress slows plant growth by modulating the GA metabolism pathway (Yamaguchi, 2008; Colebrook et al., 2014). The over-accumulation of the bioactive form GA_4_ is a new and intriguing result, suggesting that this specific GA, and not only the already known GA_3_, might play an active role in overcoming saline stress (Iqbal and Ashraf, 2013). We can speculate that the up-regulation of GA biosynthesis and active GA could be strictly linked to the lack of an ABA-mediated response to stress. Indeed, their antagonistic role under salt stress response has been recently reviewed (Ryu and Cho, 2015). Other phytohormones (brassinosteroids and ethylene) did not have a clear trend after stress, suggesting only a marginal role under our experimental conditions.

Concerning the phenolic profile (**Table 3**), the results of this study showed a clear imbalance between the different classes. Flavones and isoflavones were dissipated as a response to stress, and a similar trend was observed for almost all hydroxycinnamics. In parallel, an increase in flavonoid conjugates, namely glycosides derivatives together with flavans, was observed. Therefore, not only the total flavonoid content but also the flavonoid profile responds in a very specific manner to KCl stress.

Besides the specific and less expected metabolic changes induced by salinity stress, a large number of differential metabolites were identified because of the osmotic/oxidative stress KCl induces in plants. The detrimental effects of the increased oxidative conditions can be observed at the cytosol level, as ascorbate degradation products accumulated in KCl treated leaves. However, the results of oxidative stress toward membranes was also evident, as proven by the increase in oxidized forms of fatty acids, decrease in phospholipids, and increase in 4-hydroxy-2-nonenal that is the result of lipoperoxidation. The increase in mannitol phosphate is also consistent with the induction of osmotic stress, because of its possible role as an osmolyte.

Photosynthesis also played a major role in stress response. Biosynthetic intermediates of chlorophyllide *a* accumulated under KCl conditions in agreement with the observed increase in the SPAD index. Consistently, salinity induced the accumulation of ubiquinol and plastoquinol biosynthetic intermediates. The concurrent increase in chlorophyll catabolites suggests also a possible enhancing of chlorophyll turnover, as a response to the senescence-like effects induced by salinity stress.

## Conclusion

The results demonstrated substantial differences in the biochemical, physiological, and metabolomic responses between KCl-treated and untreated plants. Leaf dry biomass decreased in response to the application of KCl, as evidence by the fact that a significant accumulation of Cl^-^ as well as a reduction of macro and microelements in leaf tissue of artichokes was found under KCl induced-stress conditions. Regarding metabolic changes, salinity altered the hormonal network of artichokes, inducing the accumulation of auxin conjugated (inactive) form, and specifically modulating the GAs profile. The changes in the phenolic compounds profile was also a very specific response to salinity, with flavones and isoflavones reduced by KCl treatment, while flavonoid glycosides increased. Several other metabolites could be directly connected to the osmotic/oxidative stress that salinity generates (e.g., ascorbate degradation, membrane lipid peroxidation, and accumulation of mannitol phosphate). These latter results can help gain useful insight to elucidate the molecular/physiological mechanisms artichoke plants use to cope with chloride stress exposure.

## Author Contributions

LL: performed part of the metabolomic analysis, and gave an important contribution on metabolomic results interpretation; DB: followed the trial at Tuscia University, she collected the biomass and she helped on the metabolomic analysis; YR: made part of statistical analysis, and results interpretation; MC: performed the mineral analysis, statistical analysis, and results interpretation; JB: was involved in the metabolomic analysis and data interpretation; GC: defined the research idea, set up the experiment; he also made a valuable contribution on statistical analysis, results interpretation, and manuscript preparation. All authors were actively involved in writing the manuscript.

## Conflict of Interest Statement

The authors declare that the research was conducted in the absence of any commercial or financial relationships that could be construed as a potential conflict of interest. The handling Editor declared a shared affiliation, though no other collaboration, with one of the authors MC and states that the process nevertheless met the standards of a fair and objective review.

## Footnotes

1 http://www.plantcyc.org

## Supplementary Material

FIGURE S1 **Principal Component Analysis (PCA) of artichoke phenolic metabolites in KCl stressed and control plants**.

FIGURE S2 **Unsupervised hierarchical cluster analysis of artichoke metabolites in KCl stressed and control plants**.

DATA SHEET 1 **Raw data from metabolomic analysis (phenolic compounds in the first sheet and plant metabolites in the second), together with identification scores and composite spectrum**.
