# Supplementary figures and images for: Mild Potassium Chloride Stress Alters the Mineral Composition, Hormone Network, and Phenolic Profile in Artichoke Leaves
Source: Front Plant Sci. 2016 Jun 28;7:948. doi: 10.3389/fpls.2016.00948 (PMC4923119; doi:10.3389/fpls.2016.00948)

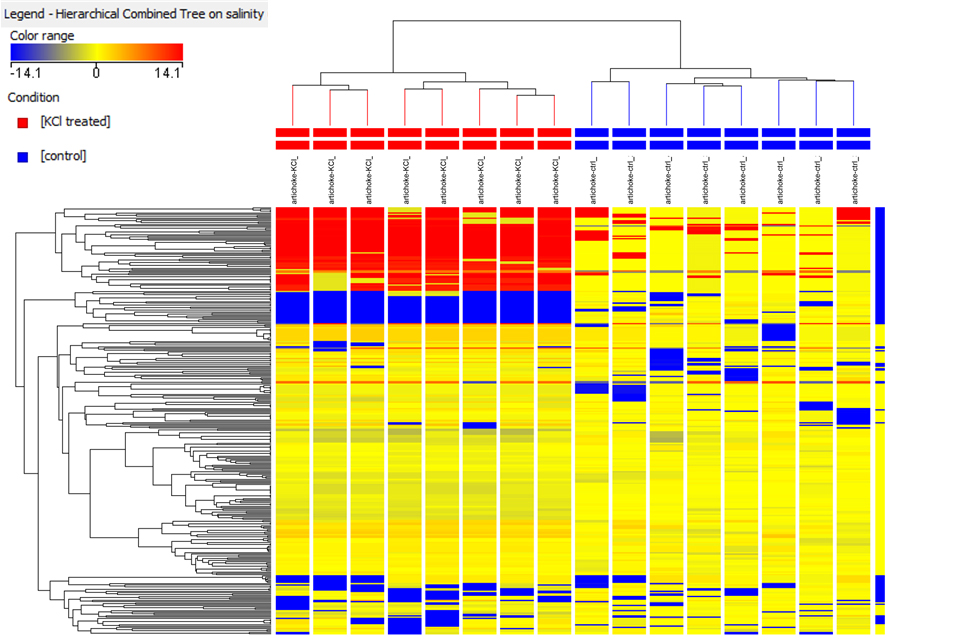

Supplement: FIGURE S2 — Unsupervised hierarchical cluster analysis of artichoke metabolites in KCl stressed and control plants. [file Image_1.JPEG]

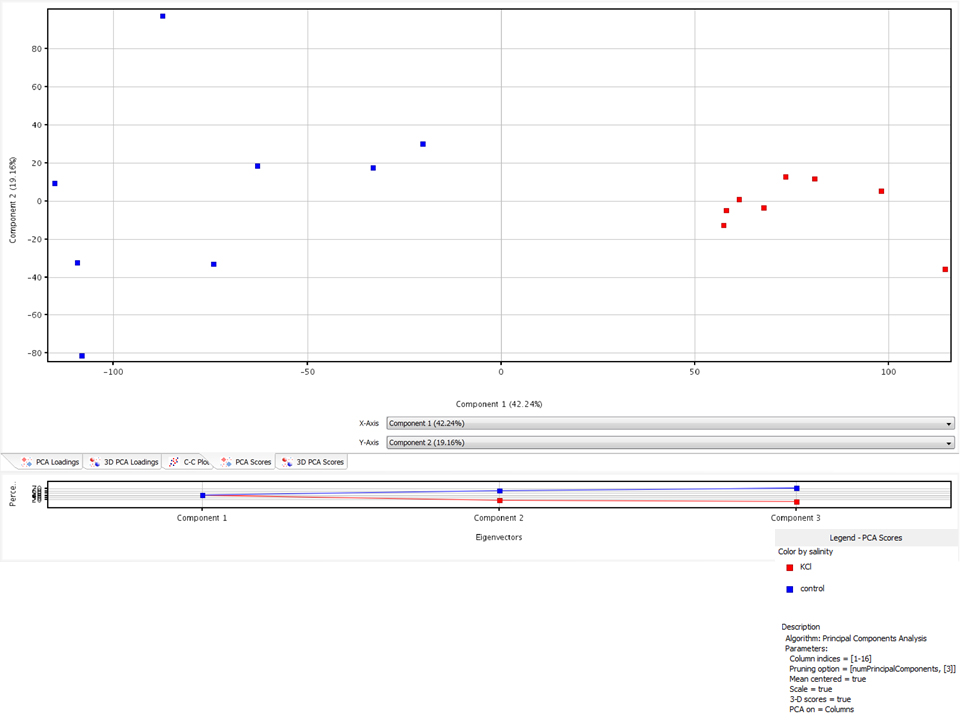

Supplement: DATA SHEET 1 — Raw data from metabolomic analysis (phenolic compounds in the first sheet and plant metabolites in the second), together with identification scores and composite spectrum. [file Image_2.JPEG]
